# A Hierarchical Bayesian Mixture Model Approach for Analysis of Resting-State Functional Brain Connectivity: An Alternative to Thresholding

**DOI:** 10.1089/brain.2020.0740

**Published:** 2020-06-17

**Authors:** Tetiana Gorbach, Anders Lundquist, Xavier de Luna, Lars Nyberg, Alireza Salami

**Affiliations:** ^1^ Department of Statistics, Umeå School of Business, Economics and Statistics, Umeå University, Umeå, Sweden.; ^2^ Umeå Center for Functional Brain Imaging, Umeå University, Umeå, Sweden.; ^3^ Department of Radiation Sciences, Umeå University, Umeå, Sweden.; ^4^ Department of Integrative Medical Biology, Umeå University, Umeå, Sweden.; ^5^ Aging Research Center, Karolinska Institutet and Stockholm University, Stockholm, Sweden.; ^6^ Wallenberg Centre for Molecular Medicine, Umeå University, Umeå, Sweden.

**Keywords:** brain aging, fMRI, functional connectivity, hierarchical modeling, lognormal distribution, resting state

## Abstract

This article proposes a Bayesian hierarchical mixture model to analyze functional brain connectivity where mixture components represent “positively connected” and “non-connected” brain regions. Such an approach provides a data-informed separation of reliable and spurious connections in contrast to arbitrary thresholding of a connectivity matrix. The hierarchical structure of the model allows simultaneous inferences for the entire population as well as for each individual subject. A new connectivity measure, the posterior probability of a given pair of brain regions of a specific subject to be connected given the observed correlation of regions' activity, can be computed from the model fit. The posterior probability reflects the connectivity of a pair of regions relative to the overall connectivity pattern of an individual, which is overlooked in traditional correlation analyses. This article demonstrates that using the posterior probability might diminish the effect of spurious connections on inferences, which is present when a correlation is used as a connectivity measure. In addition, simulation analyses reveal that the sparsification of the connectivity matrix using the posterior probabilities might outperform the absolute thresholding based on correlations. Therefore, we suggest that posterior probability might be a beneficial measure of connectivity compared with the correlation. The applicability of the introduced method is exemplified by a study of functional resting-state brain connectivity in older adults.

## Introduction

Measures of functional connectivity characterize functional architecture of the human brain by quantifying statistical dependencies between neuronal activity in distinct brain regions (Friston, 2011; Biswal et al., 1995; Sporns, 2012; Smith et al., 2011). In the case of functional magnetic resonance imaging (fMRI), neuronal activity is indirectly measured by the blood oxygenation-level-dependent (BOLD) signal. The dependencies between the regions' activity are then typically evaluated by ordinary or partial correlations of the regions' BOLD signals (Friston, 2011; Smith et al., 2011).

The connectivity matrix, constructed from the correlations between all pairs of considered brain regions, includes strong positive correlations representing reliable connections, weak correlations likely representing spurious connections (e.g., noise), as well as negative correlations. There is still an ongoing debate about the nature of negative correlations of the BOLD signal, with some studies suggesting that negative correlations have a biological basis, and others reporting those correlations as pure artifacts of preprocessing (Fox et al., 2009; Murphy et al., 2009; Murphy and Fox, 2017). Therefore, a challenging step in the connectivity analyses is a sparsification of the connectivity matrix to analyze only reliable connections and to diminish the impact of the spurious connections on further inferences.

The sparsification is performed by retaining only strong correlations in the connectivity matrix. A connectivity network, where brain regions are the nodes of the network and the sparsified connectivity matrix represents edges of the network, may be further used to yield various measures of functional brain architecture, possibly using a graph theoretical framework (Bullmore and Sporns, 2009; Rubinov and Sporns, 2010; Geerligs et al., 2014).

The two most commonly used approaches to perform sparsification of the connectivity matrix are the “absolute” and “proportional” thresholding methods (van den Heuvel et al., 2017). In the absolute thresholding, the connectivity matrix is sparsified by retaining only those dependencies that exceed some predefined cutoff, for example, correlations greater than zero (Rubinov and Sporns, 2010) or statistically significant correlations with *p*-value <0.05 (Cao et al., 2014). The main flaw of this approach is that it results in a varying number of edges across individuals, which may confound some measures of functional brain architecture such as the degree centrality (van Wijk et al., 2010). In addition, this method does not consider differences in overall connectivity strength among individuals, whereby a correlation of, for example, 0.5 may represent a reliable connection for one individual but an unreliable connection for another individual with a higher overall level of connectivity (van den Heuvel et al., 2017; Geerligs et al., 2017).

The proportional thresholding method also considers the strongest dependencies but keeps the number of connections fixed across individuals, for example, the 10% largest correlations are considered as connections (van den Heuvel et al., 2017). Since the proportional thresholding alleviates the problem of varying the number of edges across individuals, induced by absolute thresholding, it is often used before graph analysis. However, this method suffers from the arbitrariness of the cutoff as well as the unrealistic assumption of the same number of true connections across individuals.

**FIG. 1.**
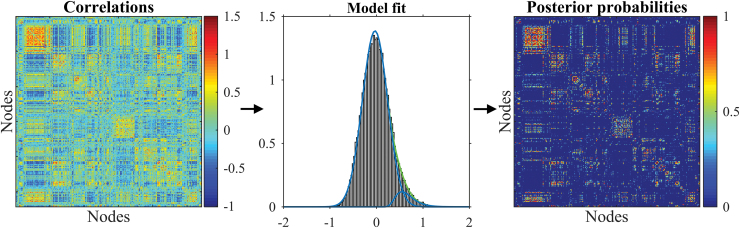
Heat map of pairwise Fisher-transformed correlations for one subject (left, axes represent brain regions, nodes), histogram of the pairwise Fisher-transformed correlations together with the model fit for the subject (center), heat map of posterior probabilities of being connected for one subject and all node pairs (right). Color images are available online.

This article aims at providing a method for the analysis of functional brain connectivity that addresses the drawbacks of the absolute and proportional thresholding methods mentioned earlier. To tackle the issue of an arbitrary cutoff in the absolute and proportional thresholding, we propose a data-informed separation of the reliable strong positive connections from the spurious correlations through mixture modeling. We assume that the distribution of the observed pairwise Fisher-transformed Pearson correlations for each subject is a mixture of two components: one for (reliably) positively connected and one for non-connected (spuriously, unreliably, as well as negatively connected) brain regions (Fig. 1). Such modeling allows for inferences about the reliable connections without explicit thresholding of a connectivity matrix through the distribution of the connected component and mixture weights.

The distributions of the mixture components are allowed to vary between individuals. Thus, the proposed approach considers the differences in the overall connectivity strength among subjects that are ignored in absolute thresholding. The mixture approach also relaxes the assumption of a constant number of connections across individuals made in proportional thresholding.

The mixture modeling of brain connectivity has been suggested in some previous studies (Chen et al., 2016; Bielczyk et al., 2018). Bielczyk et al. (2018) proposed a novel method for thresholding of a connectivity matrix based on the mixture fit. However, the connectivity was modeled separately for each subject, which restricts inferences to a subject level. Chen et al. (2016) included negative correlation in their mixture modeling approach that challenges the interpretation of the connected components given uncertainty about the nature of the negative correlation.

To face the shortcomings mentioned earlier, first, we use a lognormal distribution with positive support for the connected component to allow only positive correlations to represent the reliable connections. Second, we impose a mixed-effect structure on the distribution of the connected component. The advantage of the current approach is the possibility for simultaneous inferences on the population level via inferences on fixed effects as well as on the subject level by using the information about both fixed and random effects.

Another advantage of the mixture modeling is that it provides the posterior probability of a given pair of regions of a specific subject to be connected given the observed correlation of the regions' BOLD time series. The posterior probability, unlike correlation, takes into account individual differences in the connectivity patterns and may be seen as standardized across individuals' measure of connectivity. Thus, pairs of regions with the same observed correlation might have a different posterior probability of being connected when the subjects differ in their overall level of connectivity. This is not captured when the correlation is used as a measure of connectivity.

We also compare the use of posterior probabilities with the widespread use of correlations as measures of connectivity. We show that a significant relationship of connectivity to other measures when using the correlation may be driven by unreliable weak or negative associations, which might be alleviated by using the posterior probabilities instead. Finally, we suggest that posterior probabilities may be used for sparsification of the connectivity matrix instead of correlation. The advantages of this are shown in a simulation study.

The proposed approach is used in this article to analyze functional resting-state connectivity, its alterations in aging, and the relationship to cognition and motion by using data from the Betula project (Nilsson et al., 1997; Nilsson et al., 2004).

## Materials and Methods

### Participants

This study is based on data from 198 healthy individuals (47% females) from the Betula longitudinal project (Nilsson et al., 1997; Nilsson et al., 2004). To date, sixth waves of the Betula project have been conducted with approximately 5 years between the waves. The selected subjects entered the Betula at the first (1988–1990), second (1993–1995), and fifth (2008–2010) Betula wave, respectively, and underwent fMRI during the fifth Betula wave. These fMRI data are used in this study. The age of the participants ranged from 25 to 80 years (mean = 59, standard deviation = 13 years) at the time of scanning. The fMRI time series data from the participants were acquired at rest over a 6-min period. Participants were instructed to keep their eyes open during the scan and look at a presented fixation cross (for more detail, see Salami et al., 2014). The Regional Ethical Vetting Board at Umeå University has approved the Betula project, and all participants provided informed consent to participate.

### Imaging methods

Functional imaging was performed on a 3-T General Electric scanner equipped with a 32-channel head coil. Resting-state fMRI was acquired with a gradient echo planar imaging sequence (37 transaxial slices, thickness: 3.4 mm, gap: 0.5 mm, repetition time: 2000 ms, echo time: 30 ms, flip angle: 80°, field of view: 25 × 25 cm, 170 volumes). Before experimental image acquisition, 10 dummy scans were collected and discarded.

The fMRI data were first corrected for acquisition time differences between slices within each volume and then motion-corrected. A within-subject rigid registration was carried out to align functional and structural T1-weighted images. By means of diffeomorphic anatomical registration using exponentiated lie algebra (DARTEL; Ashburner, 2007), realigned fMRI images were nonlinearly normalized to the sample-specific group template (Salami et al., 2016), affine-aligned into stereotactic space of the Montreal Neurological Institute (MNI), and smoothed by using a 6.0-mm full width at half maximum Gaussian filter. Next, the effect of physiological noise was removed by regressing out Friston's 24 parameters of a motion model, as well as nuisance variables such as global signal, white matter, and cerebrospinal fluid signal, along with the linear trend. In addition, nuisance-corrected data were high pass-filtered (frequency >0.008 Hz).

### The analysis of fMRI data

Nodes from the Power parcellation (Power et al., 2011) were used as brain regions for functional connectivity analysis. We also added seven hippocampal and subcortical regions (10 mm diameter spheres), which were not included in the Power parcellation (MNI coordinates of centers are presented in Supplementary Table S1). As a result, 271 nodes and 36,585 initial connectivity edges per subject were included in the analysis. Time series of each node was defined as the mean of a BOLD signal over all voxels of the node.

### Cognitive measures

The cognitive tests used in this article are described in detail in Nilsson et al. (1997). Briefly, we consider four cognitive domains: episodic memory, word fluency, processing speed, and fluid intelligence. Episodic memory was measured by five tasks: immediate free recall of sentences with enactment, immediate free recall of sentences without enactment, delayed free recall of sentences with enactment, delayed free recall of sentences without enactment, and immediate free recall of unrelated nouns. Word fluency was studied by three tests where participants were asked to generate during 1 min as many words as possible starting with the letter A in the first test, five-letter words with the first letter M in the second test, and profession names with the initial letter B in the last test. Processing speed was investigated by using letter-digit substitution, letter comparison, and figure comparison tests. The Block Design test was used as an estimate of fluid intelligence. To be consistent with the previous studies where the same measures are used (Josefsson et al., 2012; Gorbach et al., 2017), the overall score for each cognitive domain was constructed as a sum of the standardized scores from the respective tests.

### Statistical model and method

We use Fisher-transformed ordinary Pearson correlation, Z_*ij*_, as a measure of dependence between the BOLD signal of nodes in pair *j* for subject $i\left(i=1,\ldots ,n;j=1,\ldots ,m\right)$. The Z_*ij*_'s are modeled as drawn from a mixture distribution of two components that represent “non-connected” and “connected” node pairs. The latent indicator variable *W*_ij_ classifies node pairs into connected or non-connected component ($W_{ij}=1$ if node pair *j* for subject *i* is connected and 0 otherwise).

To reflect the fact that only positive associations represent reliable connections, the connected component is assumed to have a lognormal distribution with support fitted on the positive real line. To model within-subject dependency of observations and between-individual differences, we introduce random effects *a_i_* and *d_i_* in the distribution of the connected component and the probability of being connected, respectively. To account for a possible shift in distribution due to the global signal regression during preprocessing, the non-connected component is assumed to have a normal distribution with the mean close to zero. Thus, the model is given by: 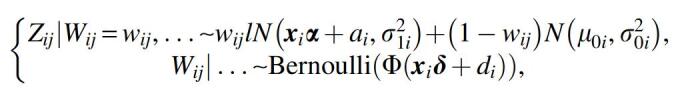


where “…” represents conditioning on covariates and the model parameters for clarity, *lN* denotes a lognormal distribution, *N* denotes a normal distribution, and φ is the cumulative distribution function of a standard normal distribution. We assume that conditionally on random effects *a_i_* and *d_i_*, and covariates$x_{i}$, $Z_{ij}$, and $W_{ij}$ are independent across individuals and node pairs. The parameters of the connected component and the probit model for *W*_ij_'s are allowed to depend linearly on a row-vector of covariates ***x****_i_* for subject *i*. In the application to the Betula project, ***x****_i_* included 1, age, sex, movement during the scan (measured as an average of framewise displacement across the scanning period (Power et al., 2012)), and cognition measures (episodic memory, word fluency, processing speed, and Block Design). Note that all covariates, except sex, were standardized to produce numerically balanced matrices. The random effects have multivariate normal distribution:

$$a_{1},\ldots ,a_{n}∼N\left(0,\gamma_{a}^{2}I_{n}\right),$$

$$d_{1},\ldots ,d_{n}∼N\left(0,\gamma_{d}^{2}I_{n}\right),$$

where $I_{n}$ is the n×n identity matrix.

We used Bayesian approach to the estimation of model parameters. Priors for α and δ were non-informative. Priors for the parameters $\mu_{0i}$ were centered around zero. We chose inverse-gamma $I\Gamma \left(1.5,10^{-3}\right)$ as a proper conditionally conjugate prior for all variance components (for generation of reasonable initial values, see the Supplementary Data). The Markov chain Monte Carlo sampling procedure and its diagnostic are described in detail in the Supplementary Data (pp. 1–10).

## Results

The model allows us to perform inference at the population level, the subject level, and the node pair level.

### Population-level analysis

**FIG. 2.**
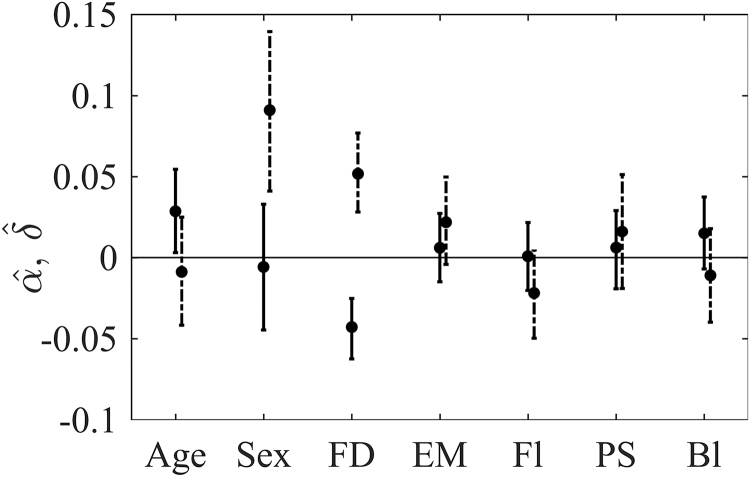
95% Credible intervals for the relationship of covariates to the strength of connections (*α*, solid line) and proportion of connections (*δ*, dashed line). Sex equals 1 for males and 0 for females. Bl, block design; EM, episodic memory; FD, framewise displacement; Fl, word fluency; PS, processing speed.

We investigated the relationship of the covariates to the distribution of the connected component and the mixing proportion , which we interpret as the proportion of connected node pairs. Results indicated that older subjects tend to have stronger connections on average (the coefficient for age $\alpha_{2}$ is estimated to be positive and 95% credible interval does not include 0), but age is not significantly related to the proportion of connections (Fig. 2).

The proportion of connections was significantly higher for men compared with women. Individuals with increased movement, measured by mean framewise displacement during the scanning, had a higher proportion of less connected node pairs. Cognitive measures did not have a significant relationship with the strength of connectivity and the proportion of observations in the connected component.

### Subject-level analysis

**FIG. 3.**
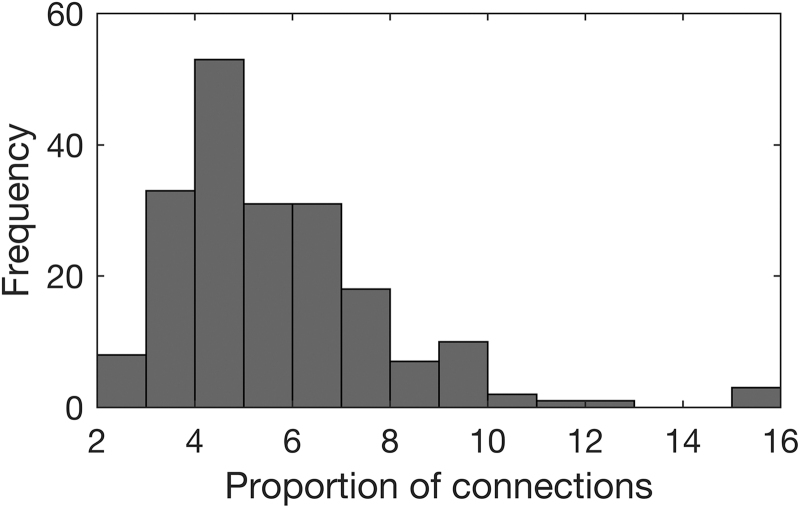
Histogram of estimated proportion of connections per subject (in %).

Subject-level inference on the proportion of connections for subject *i*, 
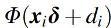
, was performed by using the posterior mean for $\sum_{j=1}^{m}W_{ij}∕m$. Figure 3 shows that the proportions of connections varied between 2% and 16% across subjects, which indicates the existence of interindividual differences and suggests that an arbitrarily chosen proportional threshold would result in too many or too little node pairs judged as connected.

### Node pair analysis

**FIG. 4.**
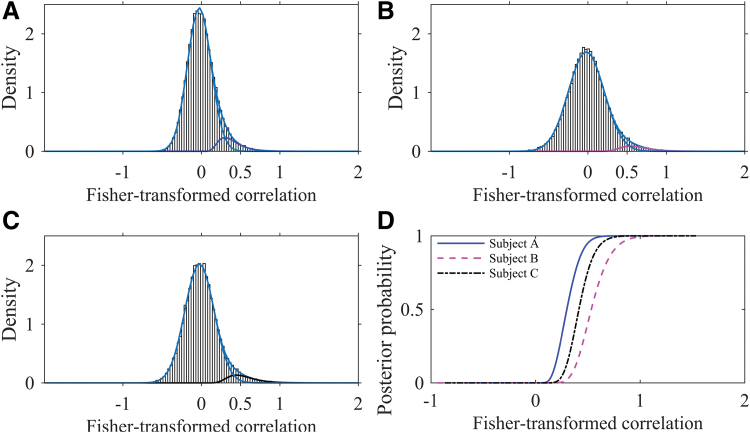
**(A–C)** Histograms of Fisher-transformed correlations for three different individuals in the Betula project on a probability density function scale with densities of fitted normal, lognormal, and mixture distributions. **(D)** Scatter plot of posterior probabilities of being connected (y-axis) versus observed correlations (x-axis), for three selected individuals. Color images are available online.

Inference can be made not only on the marginal distribution of $W_{ij}$ (as above) but also on the posterior probability of being connected for node pair *j* of subject *i* given the observed value of the correlation (posterior distribution of $W_{ij}$). The posterior probability of being in the connected component for node pair *j* of subject *i* is

$$P\left(W_{ij}=1|Z_{ij}=z_{ij},\ldots \right)=\frac{\Phi \left(x_{i}\delta +d_{i}\right)f_{lN}\left(z_{ij},x_{i}\alpha +a_{i},\sigma_{1i}^{2}\right)}{\Phi \left(x_{i}\delta +d_{i}\right)f_{lN}\left(z_{ij},x_{i}\alpha +a_{i},\sigma_{1i}^{2}\right)+\left(1-\Phi \left(x_{i}\delta +d_{i}\right)\right)f_{N}\left(z_{ij},\mu_{0i},\sigma_{0i}^{2}\right)},$$

where $f_{lN}\left(z_{ij},x_{i}\alpha +a_{i},\sigma_{1i}^{2}\right)$ and $f_{N}\left(z_{ij},\mu_{0i},\sigma_{0i}^{2}\right)$ are probability density functions of $lN\left(x_{i}\alpha +a_{i},\sigma_{1i}^{2}\right)$ and $N\left(\mu_{0i},\sigma_{0i}^{2}\right)$ distributions evaluated at $z_{ij}$ (see pp. 10–12 of the Supplementary Data for the explanation). The posterior probability of being connected for node pair *j* of subject *i* was estimated in this study by its posterior mean. We suggest that the posterior probability may be used as an alternative measure of connectivity between node pairs (see section “Validation and Comparison with Other Approaches”). As can be seen from Figure 4, different levels of correlation may correspond to the same level of posterior probability for different people as well as the same correlation may correspond to varying levels of posterior probability. This happens since location and shape of the connected component depends on the individual. For example, for some individuals, a correlation of 0.5 may be observed for many unreliable connections (Fig. 4B); whereas for other individuals, mainly reliably connected node pairs have such correlation (Fig. 4A).

**FIG. 5.**
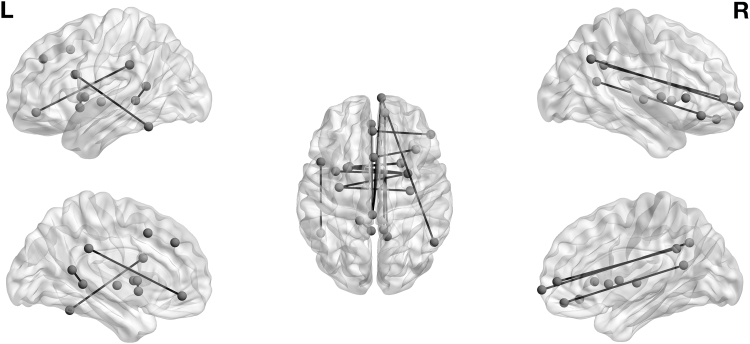
The brain graph with edges corresponding to the node pairs with significant association between age and the posterior probability of being in the connected component.

Importantly, the posterior probabilities of a connection can be related to observed covariates, such as age, sex, and cognition. For this purpose, we fitted linear regressions of posterior probabilities on the covariates of interest (for each node pair the linear regression fit is based on 198 observations that correspond to the 198 subjects included in the study). Results are summarized in Table 1: For 18 node pairs, the posterior probability of being connected was significantly negatively associated with age (after Bonferroni correction for 36,585 comparisons, Fig. 5, Supplementary Fig. S8). Five node pairs had significantly higher probability of being connected for males than females (Supplementary Table S2). Movement was significantly related to the posterior probability of being connected for 368 node pairs. The connectivity of only one node pair was significantly associated with processing speed. Other cognitive domains were not related with connectivity of specific node pairs.

**Table 1.** Number of Nodes with Significant Association (Bonferroni Corrected) Between Covariates and the Correlation or Posterior Probability of a Connection

Connectivity measure/covariate	Age	Sex	FD	EM	Fl	PS	Bl
Correlation	155	10	60	0	0	8	2
Posterior probability	18	5	368	0	0	1	0

Bl, block design; EM, episodic memory; FD, framewise displacement; Fl, word fluency; PS, processing speed.

## Validation and Comparison with Other Approaches

### Correlations versus posterior probabilities

Correlation analysis is a common approach to study functional brain connectivity (Biswal et al., 1995; Betzel et al., 2014; Wang et al., 2012). However, even if the correlation between time series of a specific node pair is unaltered with age, this might represent changes in connectivity when, for example, the connection strength of other node pairs changes. As shown in Supplementary Figure S7, the posterior probability might capture such changes.

**FIG. 6.**
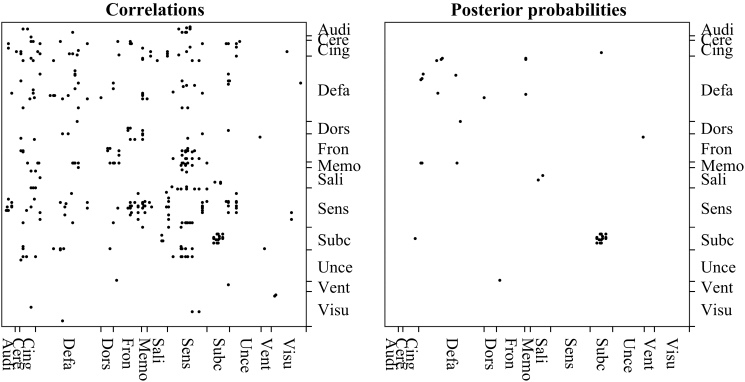
Left: node pairs, grouped by Power, 2011, network, where the correlations are significantly associated with age (Bonferroni corrected for 36,585 comparisons). Right: node pairs, grouped by network, where the posterior probabilities are significantly associated with age (Bonferroni corrected for 36,585 comparisons). Audi, Auditory; Cere, Cerebellum; Cing, Cingulate; Defa, Default mode network; Dors, Dorsal Attention; Fron, Fronto-parietal; Memo, Memory; Sali, Salience; Sens, Sensorimotor; Subc, Subcortical; Unce, Uncertain; Vent, Ventral attention; Visu, Visual.

As a comparison, we contrasted the results from the regression analyses of posterior probability against age presented earlier with those obtained by regressing Fisher-transformed correlation against age. As can be seen from Figure 6 (and Table 1), there are many more node pairs with significant age effect on the correlation than on the posterior probability (at 5% level, Bonferroni corrected).

**FIG. 7.**
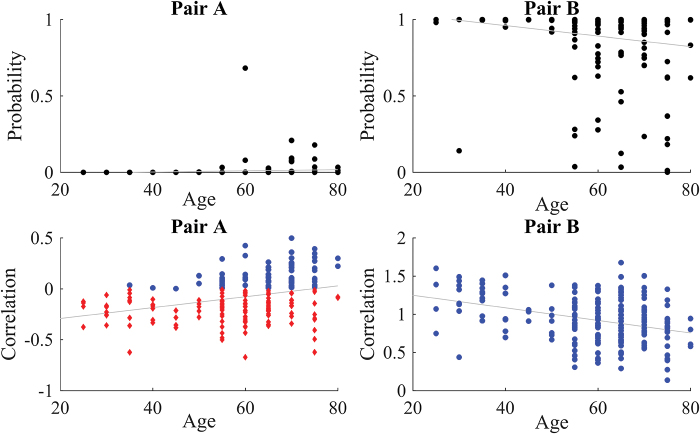
Examples of node pairs that have a significant relationship of correlation between the blood oxygenation-level-dependent signal of their nodes and age but not between posterior probability and age. Top row: scatterplot of age versus posterior probability, bottom row: scatterplots of age versus Fisher-transformed correlation; observations that have zero (red diamonds) and positive (blue circles) posterior probability of being connected. Left: node pair 30–177 from Power parcellation, with mostly negative or small positive correlations; right: node pair 145–146 with positive posterior probabilities for 197 out of 198 subjects. Color images are available online.

This difference may be due to the different contribution of negative and small positive correlations to the association with age. When Fisher-transformed correlations are considered, each correlation contributes with its strength (Fig. 7, left panel). On the other hand, when posterior probabilities are considered, all negative associations contribute with value zero (since posterior probabilities of all negative correlations are zero), and close to zero positive associations contribute with very small posterior probability. In such a way, negative and weak positive associations have a more similar contribution to association with age when posterior probabilities are considered compared with the case when Fisher-transformed correlations are studied.

In addition, even if the correlation significantly decreases with age, the posterior probability might remain high and can be less related to age (Fig. 7, right panel). At the same time, there were more node pairs that have a significant relationship between motion and posterior probabilities than between motion and correlations. The significance of the relationship in linear regression may be driven by a small number of observations with high posterior probability of being connected when most posterior probabilities are close to 0 for a node pair at hand. Therefore, when the relationship between posterior probabilities and covariates is of prior interest, one should examine the scatterplots to capture such situations as well as consider robust for outliers regressions or generalized linear models.

### A validation simulation study

We have used the simulated data set 4 from Smith et al. (2011) to compare the performance of absolute and proportional thresholding based on correlations and posterior probabilities. Briefly, the BOLD signal time series were generated for 50 individuals and 50 nodes using dynamic causal modeling. We binarized the true network matrix (A in Smith et al., 2011 with zero entries in A considered as non-connected and non-diagonal non-zero as connected brain pairs. We do not use the diagonal entries in A, which correspond to the self-associations in our analyses). As a result, each individual had 4.98% of true by definition connections. Note that the proportions of connections are the same for all subjects, which is optimal for the proportional thresholding. Our model allows for more complicated situations when the number of connections is different across subjects. We then estimated the binary true network by using absolute thresholding at 10^–6^ based on correlations or posterior probabilities; the threshold defined in Bielczyk et al. (2018) using pseudo-false discovery rate of 5%, computed separately for each individual; and proportional thresholds at 5% and 10% levels using correlations and probabilities. For all the methods, node pairs that exceed the threshold were identified as connected, and non-connected otherwise.

The results were compared in terms of the proportion of true connections that were correctly identified as such (true positive rate), proportion of true non-connected pairs that are identified as connections (false positive rate), proportion of true connections out of all node pairs identified as connected (positive predictive value), and mean proportion of correctly identified as connected or non-connected node pairs across individuals (mean performance).

We use thresholding at 10^–6^ as an approximation of thresholding at zero since small positive associations may have negligible, but still positive probability of a connection.

**FIG. 8.**
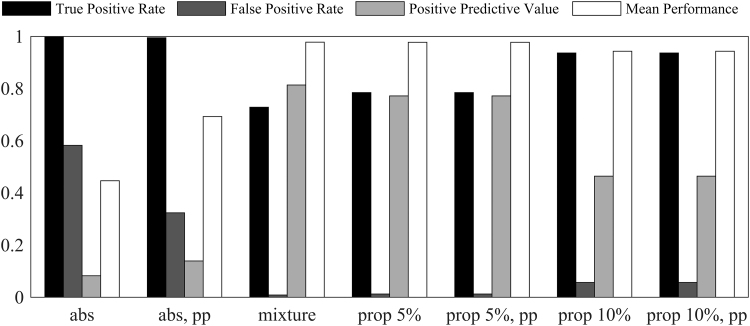
A comparison of performance of absolute thresholding at 10^−6^ based on correlation (abs), absolute thresholding at 10^−6^ based on posterior probability (abs, pp), proposed mixture modeling and thresholding based on pseudo-false discovery rate (mixture), proportional threshold based on top 5% correlations (prop 5%) and on top 5% posterior probabilities (prop 5%, pp), and proportional threshold based on top 10% correlations (prop 10%) and on top 10% posterior probabilities (prop 10%, pp).

As shown in Figure 8, absolute thresholding at 10^–6^ based on posterior probability outperforms absolute thresholding based on correlations in terms of false positive rate, positive predictive value, and mean performance. True positive rate is unsurprisingly high for the absolute thresholding at 10^–6^ based on correlations since according to this method approximately half of the node pairs are defined as connected, which also results in a high false positive rate.

The mean performance of the herein proposed mixture modeling and thresholding based on pseudo-false discovery rate is on the same level as of proportional thresholding at 5% (i.e., using approximately the true proportion of connected node pairs), whereas mixture modeling is more conservative in defining node pairs as connected and outperforms the proportional thresholding in terms of positive predictive value and false positive rate.

## Discussion

This article proposes a method for the analysis of functional brain connectivity without the explicit thresholding of a connectivity matrix. The correlations between the BOLD signals of distinct brain regions for each subject are assumed to come from a mixture of two components reflecting reliable and unreliable connections (Fig. 1). Such mixture modeling provides a data-informed separation of reliable and spurious connections in contrast to the arbitrary cutoffs used in absolute or proportional thresholding of a connectivity matrix.

Since the mixture distribution is allowed to vary across individuals (Fig. 4, for example), the presented approach permits for differences in connectivity strength between individuals, a critically hidden factor that is ignored when the absolute thresholding is applied. Moreover, the current method does not impose the unrealistic assumption of the fixed number of connections across individuals, made under the proportional thresholding.

A beneficial feature of the current model is a hierarchical structure of the distribution of the connected component. The model has parameters on the two hierarchical levels—subject-wise *a_i_* and *d_i_* and the population-level parameters α and δ. Such modeling allows inferences on the strength and proportion of connections on both the population and the subject level, and investigation of the relationship of the connected component distribution to covariates of interest, such as age, on the population level.

The comparison between correlation and posterior probabilities (Figs. 6 and 7) as connectivity measures showed that the usage of posterior probability might alleviate the effect of unreliable associations on inferences, which is present when the correlation is used as a connectivity measure. Another advantage of posterior probabilities over correlations becomes apparent when thresholding of a connectivity matrix has to be performed for further analysis. We aimed at providing a method for connectivity analyses without thresholding of a connectivity matrix. However, if thresholding is necessary for further analyses, we demonstrated in the validation exercise that absolute thresholding based on posterior probabilities may be superior to the absolute thresholding based on correlations. It should be noted here that since the absolute thresholding based on posterior probabilities results in a varying number of connections across individuals, this may still confound the graph measures calculated from the sparsified connectivity matrix.

Since pairs of regions with the highest correlation have the highest posterior probability of connection, the proportional thresholding based on correlation performs similar to the proportional thresholding based on posterior probabilities. However, the drawback of proportional thresholding is the requirement to choose a threshold for sparsification. The validation study shows that the thresholding based on pseudo-false discovery rate and model fit (Bielczyk et al., 2018) reconstructs the true connectivity pattern better than the proportional thresholding when the threshold in the proportional thresholding over- or underestimates the number of connections (Fig. 8). Moreover, when the proportion of connections varies between individuals, as suggested from our results in Figure 3, the thresholding based on model fit or pseudo-false discovery rate is also expected to outperform the proportional thresholding.

We use Pearson correlation as a measure of dependence between BOLD signals of nodes, which might reflect both direct coupling in activation between brain regions and their indirect connection (for example, nodes A and C in the model A←B→C, where arrows represent causal effects, are correlated due to the effect of B; Pearl, 2009). One may instead advocate using partial correlation as a measure of direct coupling between the activity of distinct nodes (for example, nodes A and C in the model A←B→C are conditionally uncorrelated when conditioning on B).

However, when calculating partial correlations between signals of a pair of regions, the adjustment is usually made on all other brain regions considered in the analysis (Marrelec et al., 2006; Smith et al., 2011). This might induce spurious correlations when a node activity is affected by the activity in other nodes (in the model A→B←C, A and C are marginally uncorrelated but correlated when conditioning on B). Thus, further research is needed to develop the rules for the choice of an optimal adjustment set when the partial correlation is used in the mixture modeling of brain connectivity. When causal relationships are of interest, one may consider analyses using, for example, Dynamic Causal Modeling.

We use a two-component mixture distribution to tease apart unreliable and weak connections from strongly coupled regions. As there is no consensus whether anticorrelations represent true physiological relationships (Fox et al., 2009) or are an artifact of the global signal regression (Anderson et al., 2010; Murphy et al., 2009), we focus on positive correlations for the connected component. First, this might be seen as implicit thresholding of a connectivity matrix, which makes our method not completely “threshold-free.” Second, we do not consider the existence of a component of anticorrelated regions. This is supported by visual inspection of the subject-wise distribution of the Fisher-transformed correlations that suggests the presence of positively connected component due to the clear heavy tail of the distribution on the positive side but does not indicate the existence of the anticorrelated component due to the absence of such a tail on the negative side of the distribution (Fig. 4).

To verify our “eye-inspection” of the distributions, we fitted for each subject separately a three-component mixture of a normal distribution for non-connected pairs, lognormal distribution for positive connections, and lognormal distribution mirrored on the negative side for anticorrelations (without hierarchical structure, using maximum likelihood estimation in package bbmle (Bolker and R Development Core Team, 2020) within R [R Core Team, 2018]). The results of modeling supported our initial “eye-inspection” and did not suggest the existence of the component of anticorrelated brain regions with consistent interpretation across subjects in the sample.

Note that the current modeling was applied to the data preprocessed with global signal regression. The global signal regression may help to remove global trends from the BOLD signal. In addition, the global signal regression may enhance the correspondence of functional connectivity to the structural one (Murphy and Fox, 2017). However, functional connectivity may also be studied by using the data not corrected for the global signal. In such data, most correlations of BOLD signals within a subject are expected to be positive (Fox et al., 2009; Murphy et al., 2009), and noise might require modeling by a different from a normal distribution with a mean close to zero distribution. Therefore, the mixture of normal and lognormal distribution might be a suboptimal choice for modeling of the data based on preprocessing without global signal regression.

We address the issue of spatial dependencies in the BOLD signal by considering larger nodes as brain areas, which is expected to decrease the dependencies between the nearby areas as compared with voxel-wise analysis. In addition, by using the random-effects structure in the distribution of connected component we allow for a correlation between BOLD signals of the nodes in a connected component.

The analysis of the cross-sectional data from the Betula project indicated that older subjects have on average stronger connections that might differ from some earlier studies (see Grady, 2017 for a review) but confirms the published finding of the increased magnitude of positive correlations with age, especially when it comes to between-network connectivity (Ferreira et al., 2016). Previous research showed gender-related differences in connectivity amplitude (Allen et al., 2011; Biswal et al., 2010), and our results also suggested gender-related differences in the number of connected brain regions. In addition, a significant relationship between movement and strength and proportion of connections (Fig. 2) supported the earlier suggestions of the residual relationship between connectivity and subject motion even after the movement correction during the preprocessing (Power et al., 2012; Yan et al., 2013).

We failed to find a significant Bonferroni-corrected relationship between the measures of connectivity, such as the average strength of connectivity and the proportion of observations in the connected component, and the cognition. This might be due to the global nature of the analyzed connectivity with age properties that might be not sensitive to cognitive changes. Finally, the analysis indicated a significant decrease in connectivity with age for a cluster of nodes within putamen. Interestingly, this conclusion holds when both correlation and posterior probability are used as connectivity measures. This is in line with previous findings suggesting overall lower within-network connectivity with advancing aging (Cao et al., 2014; Chan et al., 2014). However, it remains unclear why the strongest age-related difference in connectivity was found within the subcortical regions.

Finally, it is important to note that the cross-sectional nature of this study of aging is probably sensitive to cohort effects, and the method will be applied to longitudinal data in future studies.

## Conclusion

The sparsification of the connectivity matrix is an important yet challenging step in the brain connectivity analyses and is addressed in this article. Instead of an arbitrary cutoff used in traditional thresholding methods, we proposed a mixture model of observed correlations that utilizes the data for separation of reliable and unreliable connections. We presented how the novel mixed-effect structure of the mixture distribution may be used for inferences on the strength of connections and proportions of connections at the population and subject levels. We also demonstrated that the posterior probability of a connection might be a better connectivity measure compared with correlation when it comes to exploring node-pair connectivity in relation to other variables.

## Supplementary Material

Supplemental data
