# Supplementary material for: A Hierarchical Bayesian Mixture Model Approach for Analysis of Resting-State Functional Brain Connectivity: An Alternative to Thresholding
Source: Brain Connect. 2020 Jun 17;10(5):202–11. doi: 10.1089/brain.2020.0740 (PMC7310299; doi:10.1089/brain.2020.0740)
Supplement: Supplemental data [file Supp_Data.pdf]

Supplementary Data

Detailed Description of the Estimation Procedure

Specification of priors and initial values

The following priors have been considered:

$$\boldsymbol{\alpha} = (\alpha_1, \ldots, \alpha_8)' \sim N(0, 10^3 \mathbf{I}_8),$$

$$\boldsymbol{\delta} = (\delta_1, \ldots, \delta_8)' \sim N(0, 10^3 \mathbf{I}_8),$$

$$(\mu_{01}, \ldots, \mu_{0n})' \sim N(0, \gamma_0^2 \mathbf{I}_n),$$

$$\sigma_{1i}^2 \sim I\Gamma(1.5, 10^{-3}),$$

$$\sigma_{0i}^2 \sim I\Gamma(1.5, 10^{-3}),$$

$$\gamma_a^2 \sim I\Gamma(1.5, 10^{-3}),$$

$$\gamma_d^2 \sim I\Gamma(1.5, 10^{-3}),$$

$$\gamma_0^2 \sim I\Gamma(1.5, 10^{-3}).$$

The choice of prior distributions for the variance components was justified as follows: The scale parameter of inverse-gamma was chosen in accordance to the usually used non-informative $I\Gamma(10^{-3}, 10^{-3})$ prior for variances. The difference in the shape was dictated purely by the choice of initial values for the Markov chain Monte Carlo (MCMC) algorithm. As we generated initial values from the prior, traditional $I\Gamma(10^{-3}, 10^{-3})$ might have provided unreasonably high values of the variances. In addition, the scale parameter in the simulation from the posterior (full conditionals used in the MCMC algorithm) was of the form $1.5 + k/2$ (where $k = 198$ for $\gamma_0^{-2}, \gamma_a^{-2}, \gamma_d^{-2}$, $\sum_{j=1}^{m} w_{ij}$ for σ_{1i}^{-2}, and $\sum_{j=1}^{m} (1 - w_{ij})$ for σ_{0i}^{-2}), which was much higher than 1.5, so that the influence of 1.5 on the posterior vanishes. In such a way, we constructed a prior that produces reasonable initial values for the MCMC algorithm.

The initial value for α_1 was -0.75, which is the mean over all subjects of log-transformed 10% highest Fisher-transformed correlations per subject. The initial value for δ_1 was -1.28 to reflect the fact that around 1 out of 10 of node pairs are connected. Initial values for α_k, δ_k, $k = 2, \ldots, 8$ are zero. Initial values for all other parameters were drawn randomly from their priors.

Details of the Gibbs sampler

The MCMC algorithm was applied to estimate the parameters of the model. Since the prior distributions introduced in the model are conditionally conjugate, we used Gibbs sampling.

At each iteration of the MCMC, we updated the indicators of being in the connected component W_{ij}, $i = 1, \ldots, n$; $j = 1, \ldots, m$ first. This was followed by an update of γ_a^2, γ_d^2, γ_0^2, μ_{0i}, $i = 1, \ldots, n$, $\boldsymbol{\alpha}$, $a_i, i = 1, \ldots, n$, and the parameters of the probit model $\boldsymbol{\delta}$, d_i, $i = 1, \ldots, n$. The variance parameters σ_{1i}^2 and σ_{0i}^2, $i = 1, \ldots, n$ were updated last. The following full conditionals were used at each MCMC iteration:

$$W_{ij} \sim \text{Bernoulli}\left(\frac{\Phi(\boldsymbol{x}_i\boldsymbol{\delta} + d_i) f_{lN}(z_{ij}, \boldsymbol{x}_i\boldsymbol{\alpha} + a_i, \sigma_{1i}^2)}{\Phi(\boldsymbol{x}_i\boldsymbol{\delta} + d_i) f_{lN}(z_{ij}, \boldsymbol{x}_i\boldsymbol{\alpha} + a_i, \sigma_{1i}^2) + (1 - \Phi(\boldsymbol{x}_i\boldsymbol{\delta} + d_i)) f_N(z_{ij}, \mu_{0i}, \sigma_{0i}^2)} \right),$$

$$\gamma_a^{-2} \sim \Gamma\left(1.5 + \frac{n}{2}, \left(10^{-3} + \frac{\sum_{i=1}^{n} a_i^2}{2}\right)^{-1}\right),$$

$$\gamma_d^{-2} \sim \Gamma\left(1.5 + \frac{n}{2}, \left(10^{-3} + \frac{\sum_{i=1}^{n} d_i^2}{2}\right)^{-1}\right),$$

$$\gamma_0^{-2} \sim \Gamma\left(1.5 + \frac{n}{2},\ \left(10^{-3} + \frac{\sum_{i=1}^{n} \mu_{0i}^2}{2}\right)^{-1}\right),$$

where $\Gamma(k, \theta)$ is a gamma distribution with shape parameter k and scale parameter θ. The parameters $\mu_{0i}, i = 1, \ldots, n$ were updated separately by using the full conditionals:

$$\mu_{0i} \sim N\left(\psi_{\mu_{0i}} \frac{\sum_{j:w_{ij}=0} z_{ij}}{\sigma_{0i}^2}, \psi_{\mu_{0i}}\right), \text{ where } \psi_{\mu_{0i}} = \left(\frac{1}{\gamma_0^2} + \frac{\sum_{j:w_{ij}=0} (1 - w_{ij})}{\sigma_{0i}^2}\right)^{-1}.$$

The parameters in the probit model were updated by using the algorithm from Albert and Chib (1993). First, we redefined the random variables W_{ij} as follows:

$W_{ij} = 1$ if $Y_{ij} > 0$ and 0 otherwise, where Y_{ij} are independent latent variables distributed as $N(\boldsymbol{x}_i\boldsymbol{\delta} + d_i, 1)$.

The conditional distribution of Y_{ij} given $w_{ij}, \boldsymbol{\delta}, d_i, \boldsymbol{x}_i$ is:

$Y_{ij} | w_{ij}, \boldsymbol{x}_i, \boldsymbol{\delta}, d_i \sim N(\boldsymbol{x}_i\boldsymbol{\delta} + d_i, 1)$ truncated at the left by 0 if $w_{ij} = 1$,

$Y_{ij} | w_{ij}, \boldsymbol{x}_i, \boldsymbol{\delta}, d_i \sim N(\boldsymbol{x}_i\boldsymbol{\delta} + d_i, 1)$ truncated at the right by 0 if $w_{ij} = 0$.

We simulated draws from the truncated normal distribution by using the algorithm from Devroye (1986, pp. 38–39). The cumulative distribution function of the $N(\boldsymbol{x}_i\boldsymbol{\delta} + d_i, 1)$ distribution truncated at the left by 0 may be written as

$$G(y) = \begin{cases} 0, & y < 0, \\ \dfrac{F_N(y, \boldsymbol{x}_i\boldsymbol{\delta} + d_i,\ 1) - F_N(0, \boldsymbol{x}_i\boldsymbol{\delta} + d_i,\ 1)}{1 - F_N(0, \boldsymbol{x}_i\boldsymbol{\delta} + d_i,\ 1)}, & y \geq 0, \end{cases}$$

where $F_N(y, \boldsymbol{x}_i\boldsymbol{\delta} + d_i,\ 1)$ is the value of the cumulative distribution function of $N(\boldsymbol{x}_i\boldsymbol{\delta} + d_i,\ 1)$ distribution evaluated at y. Then, $Y_{ij} | w_{ij}, \boldsymbol{x}_i, \boldsymbol{\delta}, d_i \sim N(\boldsymbol{x}_i\boldsymbol{\delta} + d_i, 1)$ truncated at the left by 0 may be generated as $F_N^{-1}(F_N(0, x_i\boldsymbol{\delta} + d_i,\ 1) + U * (1 - F_N(0, \boldsymbol{x}_i\boldsymbol{\delta} + d_i,\ 1)), \boldsymbol{x}_i\boldsymbol{\delta} + d_i,\ 1)$, where U is a uniform random variable on [0,1] and $F_N^{-1}(y, \boldsymbol{x}_i\boldsymbol{\delta} + d_i,\ 1)$ denotes an inverse of the cumulative distribution function of $N(\boldsymbol{x}_i\boldsymbol{\delta} + d_i,\ 1)$ distribution evaluated at y.

In a similar way, the distribution of $Y_{ij} | w_{ij}, \boldsymbol{x}_i, \boldsymbol{\delta}, d_i \sim N(\boldsymbol{x}_i\boldsymbol{\delta} + d_i, 1)$ truncated at the right by 0 may be written as

$$G(y) = \begin{cases} \dfrac{F_N(y, \boldsymbol{x}_i\boldsymbol{\delta} + d_i,\ 1)}{F_N(0, \boldsymbol{x}_i\boldsymbol{\delta} + d_i,\ 1)}, & y \leq 0, \\ 0, & y > 0. \end{cases}$$

Then, $Y_{ij} | w_{ij}, \boldsymbol{x}_i, \boldsymbol{\delta}, d_i \sim N(\boldsymbol{x}_i\boldsymbol{\delta} + d_i, 1)$ truncated at the right by 0 may be generated as $F_N^{-1}(U * F_N(0, \boldsymbol{x}_i\boldsymbol{\delta} + d_i,\ 1), \boldsymbol{x}_i\boldsymbol{\delta} + d_i,\ 1)$.

We proceeded with an update of $\boldsymbol{\alpha}$, $a_1, \ldots, a_n$ by using the linear model

$$\ln Z_{ij} = \boldsymbol{x}_i\boldsymbol{\alpha} + a_i + e_{ij},\ i, j : w_{ij} = 1 \tag{1}$$

and an update of $\boldsymbol{\delta}$ and $d_1, \ldots, d_n$ by using the model

$$Y_{ij} = \boldsymbol{x}_i\boldsymbol{\delta} + d_i + \epsilon_{ij},\ \ i = 1, \ldots, n, j = 1, \ldots, m, \tag{2}$$

where $e_{ij} \sim N(0, \sigma_{1i}^2)$ and $\epsilon_{ij} \sim N(0, 1)$ are independent over i and j, and they are uncorrelated with $\boldsymbol{x}_i$. Here, w_{ij} is the value of W_{ij} at the current MCMC iteration. The algorithm is the same for $\boldsymbol{\alpha}$, $a_1, \ldots, a_n$ and $\boldsymbol{\delta}$, $d_1, \ldots, d_n$.

After numerous attempts to run estimation by using different algorithms (among others, update one parameter at a time; update fixed effects separately from the random effects with random effects updated together; and update fixed effects separately from the random effects with random effects updated one at a time), updating fixed and random effects simultaneously in the linear models provided the quickest convergence. Therefore, we updated fixed and random effects together in each MCMC iteration.

Linear model (Eq. 2) is a particular case of the general Bayesian model (see Bryk and Raudenbush, 1992, Chapter 10).

$$\boldsymbol{Y} = \boldsymbol{A\theta} + \boldsymbol{r},$$

where $\boldsymbol{r} \sim \boldsymbol{N}(\boldsymbol{0},\ \Psi)$, $\boldsymbol{Y} = \begin{pmatrix} Y_{11} \\ \ldots \\ Y_{1m} \\ Y_{21} \\ \ldots \\ Y_{2m} \\ \ldots \\ Y_{nm} \end{pmatrix}$, $\boldsymbol{A} = \begin{pmatrix} \boldsymbol{x}_1 & 1 & 0 & \ldots & 0 \\ \ldots & 1 & 0 & \ldots & 0 \\ \boldsymbol{x}_1 & 1 & 0 & \ldots & 0 \\ \boldsymbol{x}_2 & 0 & 1 & \ldots & 0 \\ \ldots & 0 & 1 & \ldots & 0 \\ \boldsymbol{x}_2 & 0 & 1 & \ldots & 0 \\ \ldots & 0 & 0 & \ldots & 0 \\ \boldsymbol{x}_n & 0 & 0 & \ldots & 1 \end{pmatrix}$, $\boldsymbol{\theta} = \begin{pmatrix} \delta_1 \\ \ldots \\ \delta_8 \\ d_1 \\ \ldots \\ d_n \end{pmatrix}$,

$\Psi = \mathbf{I_{nm}}$ is the $nm \times nm$ identity matrix. The prior distribution for $\boldsymbol{\theta}$ is $N(\mathbf{0},\ \Omega)$, where $\Omega = \mathrm{diag}\left(10^3,\ \ldots,10^3,\ \gamma_{\mathrm{d}}^2,\ \ldots,\gamma_{\mathrm{d}}^2\right)$ is an $(n+8)\times\ (n+8)$ diagonal matrix.

The parameter $\boldsymbol{\theta}$ was updated by using the full conditional distribution

$$\boldsymbol{\theta}|\boldsymbol{Y},\ \Psi,\ \Omega \sim N(\boldsymbol{\theta}^*,\ \boldsymbol{D}^*),\ \text{where}\ \boldsymbol{\theta}^* = \boldsymbol{D}^*\boldsymbol{A}'\Psi^{-1}\boldsymbol{Y},\ \boldsymbol{D}^* = \left(\boldsymbol{A}'\Psi^{-1}\boldsymbol{A} + \Omega^{-1}\right)^{-1} \tag{3}$$

The parameters of linear model (1) were updated by using the full conditionals (3) with Y_{ij} substituted by $\ln Z_{ij}$, $\boldsymbol{\theta} = (\alpha_1,\ \ldots,\ \alpha_8,\ a_1,\ \ldots,\ a_n)'$, $\Omega = \mathrm{diag}\left(10^3,\ \ldots,10^3,\ \gamma_{\mathrm{a}}^2,\ \ldots,\gamma_{\mathrm{a}}^2\right)$, $\boldsymbol{\Psi} = \mathrm{diag}\left(\sigma_{11}^2,\ \ldots,\ \sigma_{11}^2,\ \sigma_{12}^2,\ \ldots,\sigma_{12}^2,\ \ldots,\sigma_{1n}^2\right)$, and using only the rows of $\ln\boldsymbol{Z} = (\ln Z_{11},\ \ldots,\ \ln Z_{1m},\ \ldots,\ \ln Z_{nm})'$ and $\boldsymbol{A}$, which correspond to pairs defined as connected at the current iteration $\left(w_{ij} = 1\right)$.

Finally, σ_{1i}^2 and σ_{0i}^2 were updated separately for each individual using the distribution:

$$\sigma_{1i}^{-2} \sim \Gamma\left(1.5 + \frac{\sum_{j:\ w_{ij}=1} w_{ij}}{2},\ \left(10^{-3} + \frac{\sum_{j:\ w_{ij}=1}\left(\ln z_{ij} - \boldsymbol{x}_i\boldsymbol{\alpha} - a_i\right)^2}{2}\right)^{-1}\right),$$

$$\sigma_{0i}^{-2} \sim \Gamma\left(1.5 + \frac{\sum_{j:\ w_{ij}=0}\left(1 - w_{ij}\right)}{2},\ \left(10^{-3} + \frac{\sum_{j:\ w_{ij}=0}\left(z_{ij} - \mu_{i0}\right)^2}{2}\right)^{-1}\right).$$

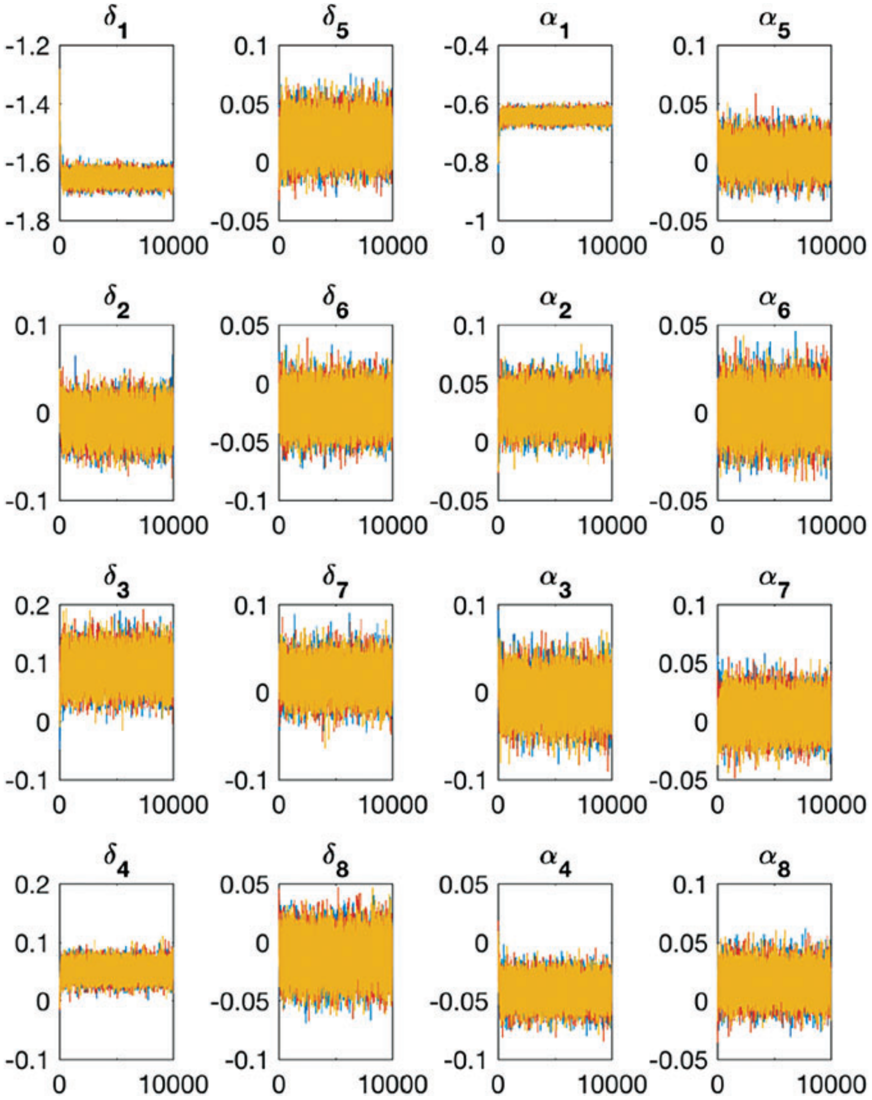

SUPPLEMENTARY FIG. S1. Trace plots for fixed effects parameters from the three MCMC chains (in blue, orange, and yellow). MCMC, Markov chain Monte Carlo.

Assessment of MCMC convergence

We ran three Markov chain sequences of 10,000 iterations (with different starting values as discussed earlier corresponding to seeds 0, 10,4651, and 30,113) by using MATLAB (2016). As can be seen from Supplementary Figure S1, the approximate convergence has been achieved.

The autocorrelation of the second half of iterations was negligible even with lag 1 for the fixed effects parameters (see examples in Supplementary Fig. S2). However, the autocorrelation remained noticeable for some of the other parameters even at lag 20 (see examples in Supplementary Fig. S3). Therefore, to compromise between sequence autocorrelations and the amount of data used for final inferences, we defined burn-in of 5000 iterations and thinned the sequences by retaining every fifth iteration after that. The estimated value of the scale reduction factor ($\hat{R}$, Gelman et al., 2003) for all parameters was <1.06. We used the mean of one thinned sequence (for a chain with seed 0) for posterior inference for the parameters of interest.

Generated Data Example

To ensure that the algorithm is able to converge close to the true values of the model parameters, we ran the estimation procedure on generated data. Fisher-transformed Pearson correlations were generated from the estimated model for 198 individuals and 10,000 node pairs. To define the values of the parameters for data generation, we first ran the analysis on the Betula data and then used the posterior means for the parameters of interest to generate data. We then ran three chains of the MCMC of 10,000 iterations on the generated data, assessed convergence, and compared the estimates for the parameters with the ones used in the data generation process. The scale reduction factor for the thinned sequences (that includes each fifth iteration after the burn-in of 5000 iterations) was estimated to be <1.06 for all parameters of the model, and the estimates were close to the generated values of the parameters (Supplementary Fig. S4). The 95% credible intervals for all fixed effects included the true values of the parameters.

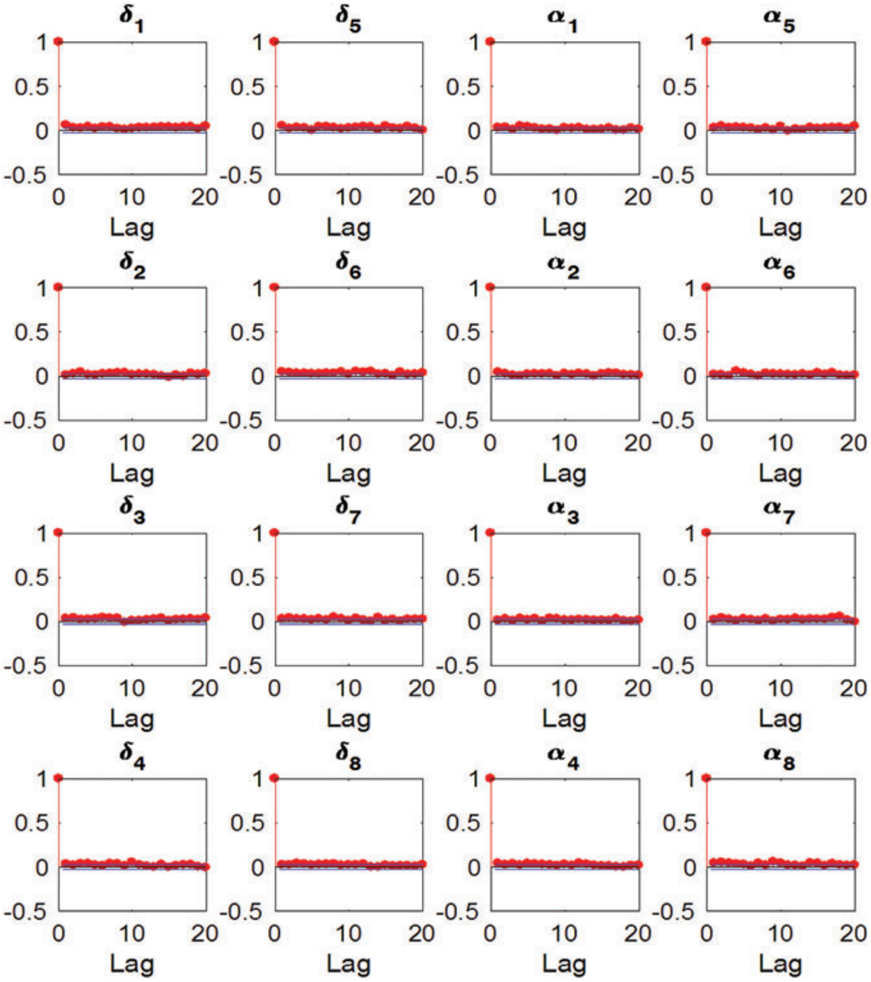

SUPPLEMENTARY FIG. S2. Autocorrelation within the iterations 5000–10,000 of the MCMC chain with seed 0 for fixed effects.

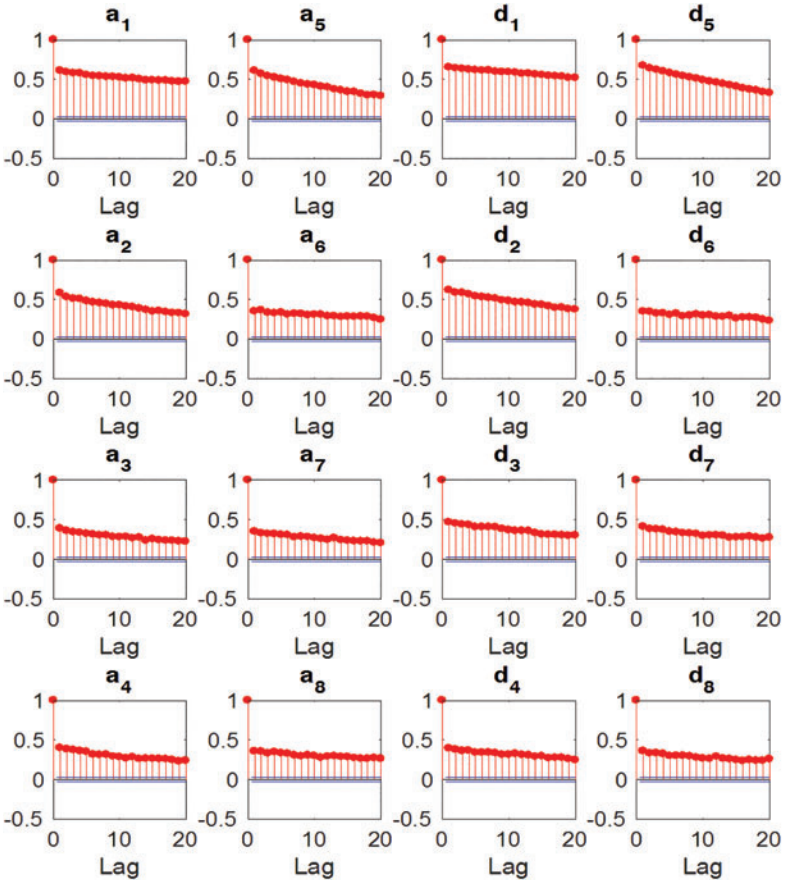

SUPPLEMENTARY FIG. S3. Autocorrelation within the iterations 5000–10,000 of the MCMC chain with seed 0 for random effects a_i, d_i, $i = 1, \ldots, 8$.

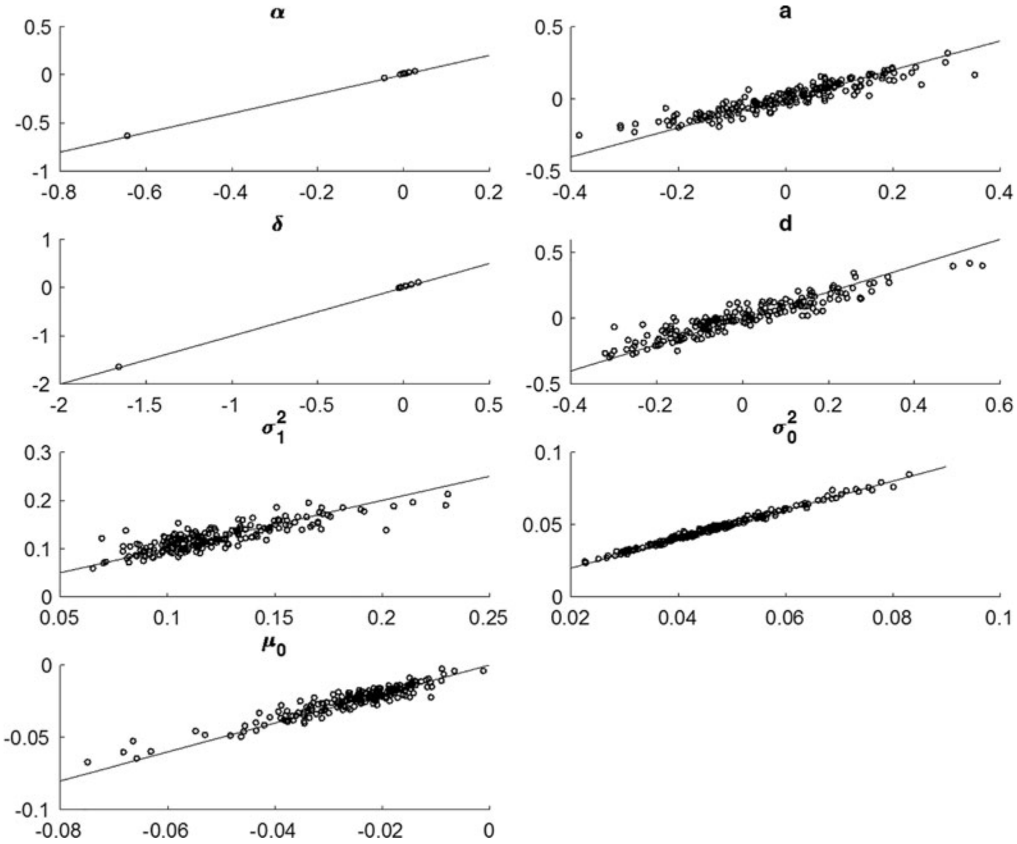

SUPPLEMENTARY FIG. S4. The scatterplots of the true generated (x-axis) versus estimated values (y-axis) of the model parameters with the line y = x indicated.

Model Fit to the Betula Data

We first examined the model fit by graphical posterior checks, and the model seemed to fit the data reasonably well. Numerical posterior predictive checks were performed as follows:

- We simulated $L = 200$ replications of the data by first selecting a draw from the posterior distribution and then using the estimated parameters from the draw to generate the replication l of the data, $y^{\text{rep},l}$;
- We computed the minimum and the maximum of the replicated data for each individual i and replication l: $\max y_i^{\text{rep},l} = \max\left(\{y_{ij}^{\text{rep},l},\ j=1,\ldots,m\}\right)$, and $\min y_i^{\text{rep},l} = \min\left(\{y_{ij}^{\text{rep},l},\ j=1,\ldots,m\}\right)$;
- We computed the proportion of the maximum values from the replications that are higher than the maximum observed value for a subject i (i.e., the posterior probability to get at least as high maximum value as the one obtained with the real data): $p_{i,\ \max} = \#\{l=1,\ldots,L:\ \max y_i^{\text{rep},l} > \max(\{z_{ij},\ j=1,\ \ldots,m\})\}/L$;
- In a similar way, we computed the posterior probability to get at least as low minimum value as the one obtained with the real data: $p_{i,\ \min} = \#\{l=1,\ldots,L:\ \min y_i^{\text{rep},l} < \min(\{z_{ij},\ j=1,\ \ldots,m\})\}/L$;

The same procedure was conducted for 99.9th, 99th percentiles (instead of a maximum) and 0.1st and 1st percentiles (instead of a minimum), respectively.

As can be seen from Supplementary Figure S5, the model fitted the data well for most individuals. The high p-values for the maximum might be due to the long tail of the lognormal distribution that resulted in simulating highly improbable, extreme observations when a lot of data were generated (for each individual and replication, 36,585 observations were generated). As expected, the distribution of p-values evened out when the 99.9th percentile (36,548th observation out of 36,585) was considered. The distribution of minimum, 0.1st, and 1st percentiles suggested that the respective quantiles were systematically higher in the replicated data than in the observed one for some individuals. This might indicate a possibility for model improvement by consideration of distribution with heavier tails for the non-connected component.

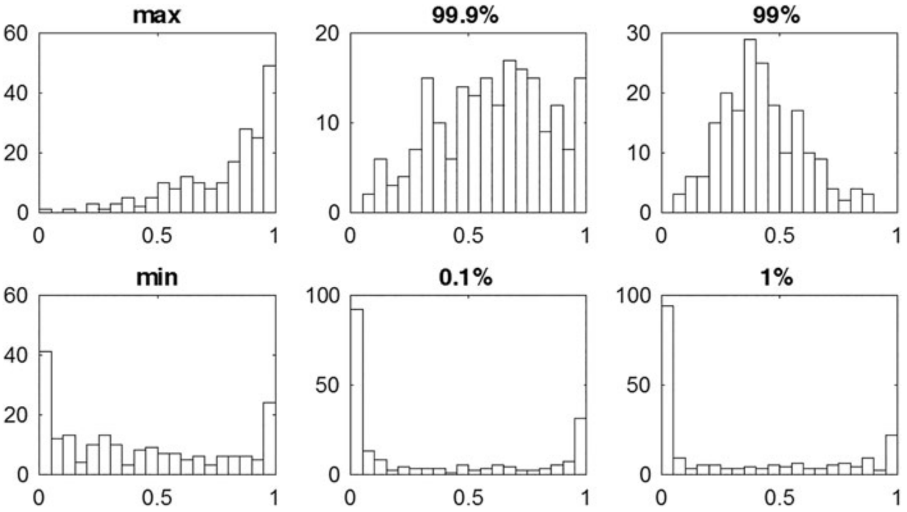

SUPPLEMENTARY FIG. S5. Top row: the distribution of $p_{i,\max}$ across subjects for the maximum on the left histogram, 99.9th percentile in the center, 99th percentile on the right. Bottom row: the distribution of $p_{i,\ \min}$ across subjects for the minimum value on the left histogram, 0.1st percentile in the center, 1st percentile on the right.

Definition and Properties of Posterior Probabilities

Marginal distribution of W_{ij}

The marginal probability of a node pair for subject i to be in the connected component is $\Phi(\boldsymbol{x}_i\boldsymbol{\delta} + d_i)$. Suppose subject i is chosen, and the researcher does not consider a specific node pair. Then, $\Phi\left(\boldsymbol{x}_i^*\boldsymbol{\delta} + d_i\right)$ gives a probability of a node pair to be connected for this person. The parameter $\boldsymbol{\delta}$ represents covariates' effect on such probability.

Conditional distribution of W_{ij}

The probability of a specific node pair j for a specific individual i to be connected given the value of the observed correlation and covariates may be expressed as follows:

$$P\left(W_{ij}=1|Z_{ij}=z_{ij},\ \ldots\right) = \frac{f\left(z_{ij}|W_{ij}=1,\ \ldots\right)P\left(W_{ij}=1|\ldots\right)}{f(z_{ij}|\ldots)}$$
$$= \frac{\Phi(\boldsymbol{x}_i\boldsymbol{\delta}+d_i)f_{lN}\left(z_{ij},\boldsymbol{x}_i\boldsymbol{\alpha}+a_i,\ \sigma_{1i}^2\right)}{\Phi(\boldsymbol{x}_i\boldsymbol{\delta}+d_i)\, f_{lN}\left(z_{ij},\boldsymbol{x}_i\boldsymbol{\alpha}+a_i,\ \sigma_{1i}^2\right) + (1-\Phi(\boldsymbol{x}_i\boldsymbol{\delta}+d_i))\, f_N\left(z_{ij},\mu_{0i},\ \sigma_{0i}^2\right)}.$$

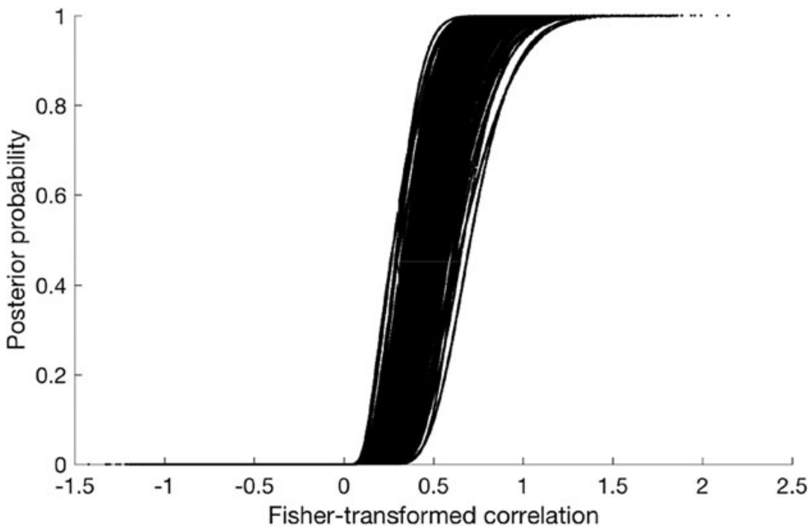

SUPPLEMENTARY FIG. S6. The scatterplot of the relationship between the observed Fisher-transformed correlations and estimated posterior probabilities of being in the connected component across individuals and node pairs of the Betula data.

As can be seen from Supplementary Figure S6, the estimated posterior probabilities generally increase with increasing correlation.

Properties of posterior probabilities

In what follows, we demonstrate the behavior of posterior probabilities when the overall distribution of the Fisher-transformed correlations changes with age. We consider the cases when the correlation for a specific node pair stays constant over time and show that changes are seen in the distribution of posterior probabilities (see Supplementary Fig. S7). For this, we use the mixture distribution, which is close to the one, fitted to a subject from Betula data, and we calculate posterior probabilities for each value of observed correlation by using the formula for $P\left(W_{ij}=1|Z_{ij}=z_{ij},\ \ldots\right)$ presented earlier.

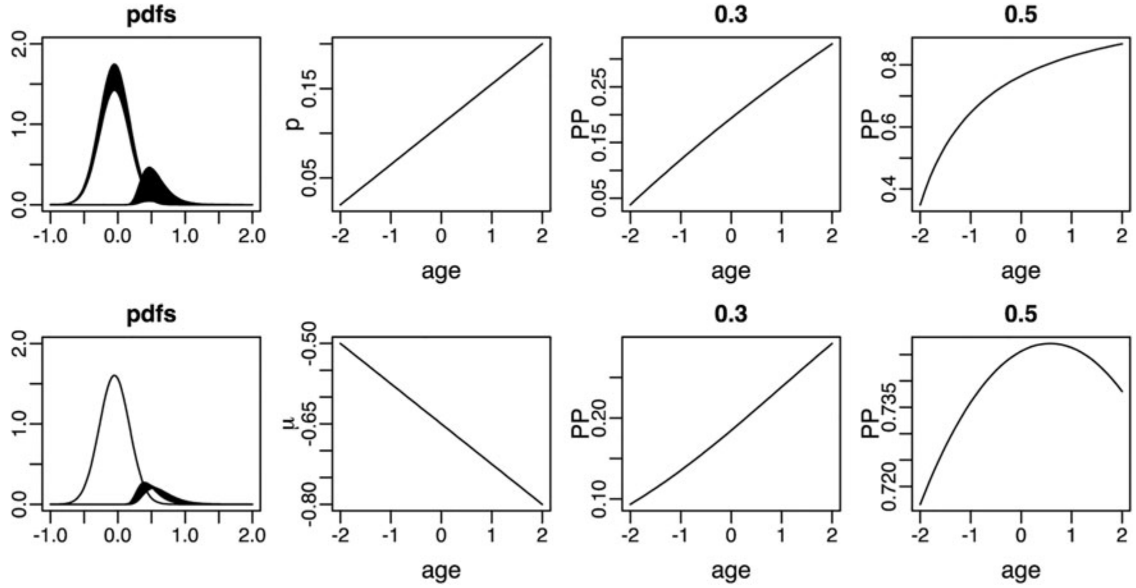

SUPPLEMENTARY FIG. S7. Top left: mixture components with varying proportion of connections (p), the other parameters of the model are unchanged. Bottom left: mixture components with varying first parameter of the lognormal distribution (μ), the other parameters of the model are unchanged. Second column from the left, top row: plot of proportion of connections p versus the generated age, bottom row: plot of μ versus the generated age. Other plots: plots of PP for a specific value of the correlation between the nodes' signal, which is indicated in the plot title, for changing p in the top row and μ in the bottom row versus age. PP, posterior probabilities.

Additional Regions Included in the Analysis

Regions 1–264 are Power nodes.

Supplementary Table S1. Montreal Neurological Institute Coordinates of Centers of Additional Regions Included in the Analysis

Index of a region	*X*	*Y*	*Z*
265	−24	6	4
266	10	14	2
267	−12	12	6
268	−28	−12	−20
269	28	−10	−22
270	−18	−26	−12
271	18	−24	−10

Node Pairs with Significant Effect of Sex on Posterior Probability

Supplementary Table S2. Node Pairs with Significant Effect of Sex on Posterior Probability

018Sens–035Sens	029Sens–041Sens	067Audi–071Audi	081Defa–248Unce	163Visu–167Visu

Scatterplots for Node Pairs with Significant Effect of Age on Posterior Probabilities

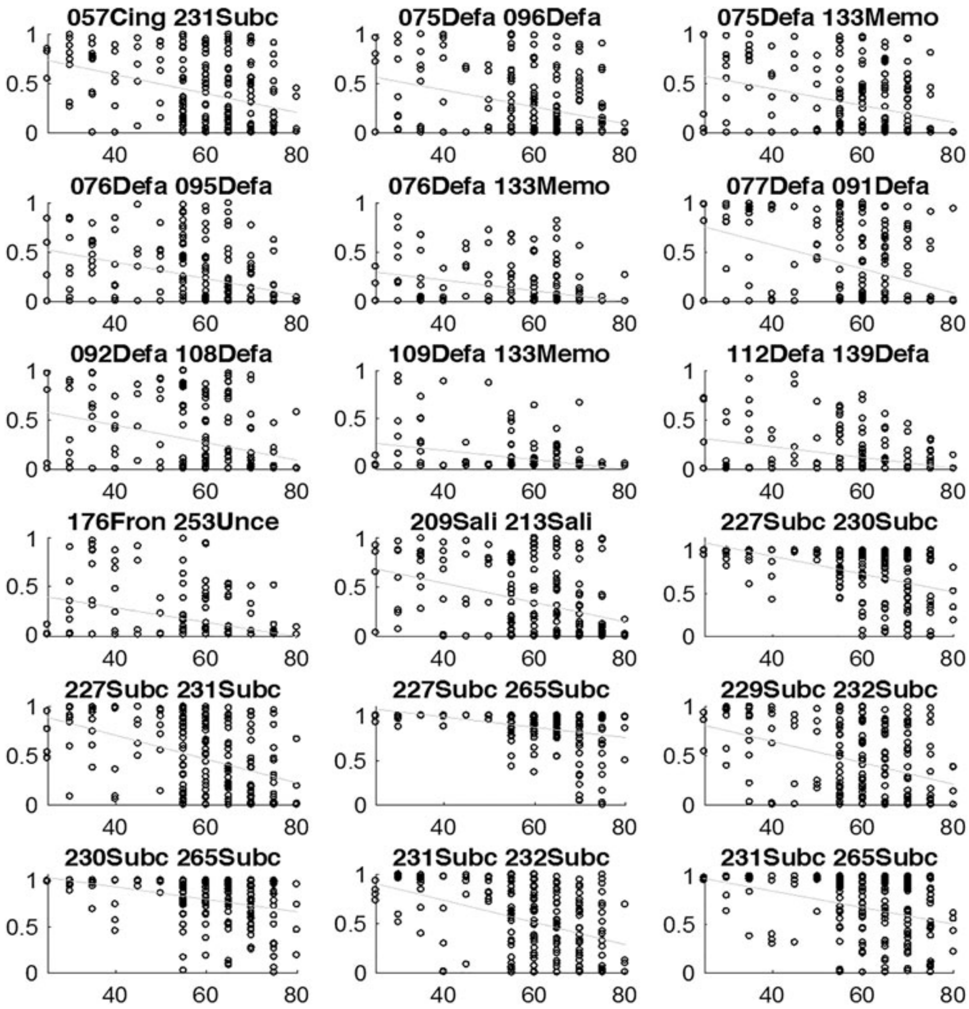

SUPPLEMENTARY FIG. S8. Scatterplots of age (x-axis) versus posterior probability of being in the connected component for 18 node pairs that display a significant effect of age on the posterior probability. The names of the nodes are represented in the title of each plot (order number in the Power parcellation [Power et al., 2011] and shortened name of the network to which the node corresponds).

Supplementary References

Albert JH, Chib S. 1993. Bayesian analysis of binary and polychotomous response data. J Am Stat Assoc 88:669–679.

Bryk AS, Raudenbush SW. 1992. *Hierarchical Linear Models: Applications and Data Analysis Methods.* Thousand Oaks, CA: Sage Publications, Inc.

Devroye L. 1986. *Non-Uniform Random Variate Generation.* New York: Springer-Verlag New York Inc.

Gelman A, Carlin JB, Stern HS, Rubin DB. 2003. *Bayesian Data Analysis*, 2nd ed. Boca Raton: Chapman and Hall/CRC.

MATLAB. 2016. *MATLAB Release 2016b*. Natick, MA: The MathWorks, Inc.

Power JD, Cohen AL, Nelson SM, Wig GS, Barnes KA, Church JA, et al. 2011. Functional network organization of the human brain. Neuron 72:665–678.
